# LC-MS and NMR Based Plant Metabolomics: A Comprehensive Phytochemical Investigation of *Symphytum anatolicum*

**DOI:** 10.3390/metabo13101051

**Published:** 2023-10-04

**Authors:** Hilal Kılınc, Gilda D’Urso, Annunziata Paolillo, Ozgen Alankus, Sonia Piacente, Milena Masullo

**Affiliations:** 1 Department of Geological Engineering, Engineering Faculty, Dokuz Eylul University, Buca, 35370 İzmir, Turkey; hilal.altunkeyik@deu.edu.tr; 2 Dipartimento di Farmacia, Università degli Studi di Salerno, Via Giovanni Paolo II, Fisciano, 84084 Salerno, Italy; gidurso@unisa.it (G.D.); mmasullo@unisa.it (M.M.); 3 Chemistry Department, Faculty of Science, Ege University, Bornova, 35100 Izmir, Turkey; ozgen.alankus@ege.edu.tr

**Keywords:** *Symphytum anatolicum*, Boraginaceae, LC-MS, NMR, α-glucosidase assay, tyrosinase assay

## Abstract

The application of metabolomics to the study of plants is growing because of the current development of analytical techniques. The most commonly used analytical technology driving plant metabolomics studies is Mass Spectrometry (MS) coupled to liquid chromatography (LC). In recent years, Nuclear Magnetic Resonance (NMR) spectroscopy, not requiring a previous chromatographic separation, has been receiving growing attention for metabolite fingerprinting of natural extracts. Herein, an integrated LC-MS and ^1^H NMR metabolomic approach provided a comprehensive phytochemical characterization of *Symphytum anatolicum* whole plant, taking into account both primary and specialized metabolites. Moreover, the NMR analyses provided direct quantitative information. Species belonging to the *Symphytum* genus, known as comfrey, have shown several biological activities including anti-inflammatory, analgesic, hepatoprotective, antifungal, and antibacterial. The LC-MS profile showed the presence of 21 main specialized metabolites, belonging to the classes of flavonoids, phenylpropanoids, salvianols, and oxylipins. The ^1^H NMR spectrum revealed the occurrence of metabolites including organic acids, phenolics, flavonoids, sugars, and amino acids. A quantitative analysis of these metabolites was performed and their concentration was obtained with respect to the known concentration of TSP, by means of the software package Chenomx which allows quantification of individual components in the NMR spectra. Furthermore, the phenolic content, antioxidant activity, glucosidase, and tyrosinase inhibitory activity of *S. anatolicum* extract were evaluated. The resulting bioactivity profile suggests how *S. anatolicum* represents a source of metabolites with health-promoting activity.

## 1. Introduction

Metabolomics is an *omics* approach that can be used to acquire comprehensive information on the composition of food and medicinal plants. The use of metabolomics in ecology is more recent, with some exceptions [1]. The metabolome is the quantitative collection of all the low molecular weight compounds present in a cell organism in a particular physiological or developmental state. Early studies used analytical standards to determine the presence or abundance of a limited set of pre-determined metabolites in a sample. The field has now shifted towards untargeted metabolomics, which instead attempts to detect and identify all the metabolites in a sample [1]. The two most commonly used analytical technologies driving quantitative plant metabolomics studies are Mass Spectrometry (MS) and Nuclear Magnetic Resonance (NMR) spectroscopy [2].

Due to its high sensitivity, MS is by far the technology of choice in most plant metabolomics studies especially when coupled to powerful chromatographic techniques (e.g., liquid chromatography–mass spectrometry (LC-MS)). NMR spectroscopy is largely used in the field of quality evaluation of various food and medicinal plants, and it is widely used for the characterization and structure determination of natural products. NMR spectrum can provide a wealth of accurate qualitative and quantitative information even regarding the components of a mixture [3]. In most cases, in contrast to GC-MS and LC-UV/MS techniques, the NMR approach does not require separation or isolation of analytes from a mixture. Hence, the loss of the whole metabolite information caused by the filtration of chromatographic columns can be avoided (such loss is very likely for botanicals since they have a wide range of distributions both of molecular weight and polarity) [4].

Both approaches provide complementary as well as supplementary information on the metabolite profiles of plants and herein they have been employed to provide a comprehensive phytochemical characterization of *Symphytum anatolicum*, an endemic Turkish plant, less investigated if compared to the well-known *Symphytum officinale* (comfrey).

The genus *Symphytum* L. (Boraginaceae) is represented by perennial plants and includes about 35 species [5,6,7]. Turkey which hosts different climatic conditions in a wide area, has a rich plant diversity and the *Symphytum* genus is represented by 18 species among which *Symphytum tuberosum* L., *Symphytum officinale* L., *Symphytum caucasicum* Bieb., and *Symphytum anatolicum* Boiss [8].

Various species of *Symphytum* have shown anti-inflammatory, analgesic, hepatoprotective, antifungal, and antibacterial activity [5,9]. Different formulations obtained from the roots of *S. officinale* are used as a topical anti-inflammatory remedy and in the treatment of arthritis [10,11].

Furthermore, *Symphytum* spp. are reported to be an excellent remedy for fractures and injuries, facilitating the joining of broken bones and reducing the pain of needling [12].

*Symphytum anatolicum* Boiss. is an ornamental and medicinal plant growing in Europe, Asia, and North America. The plant was first collected by d’Urville and named *S. asperrimum* d’Urv, then Boissier changed its name to *S. anatolicum* Boiss [8,13].

The extracts of *S. anatolicum* roots exhibited antioxidant activity and protective effects against tyrosinase and α-amylase [14], aerial parts of *S. anatolicum* showed a high inhibitory capacity, especially against acetylcholinesterase and α-glucosidase enzymes [15,16]. *S. anatolicum* is also used in wound healing and the treatment of sprains and bruises [5].

Indeed, the genus contains many important bioactive constituents, including alkaloids, flavonoids, triterpenes, tannins, sterols, polysaccharides, and amino acids [5]. Among alkaloids, pyrrolizidine-type alkaloids are found in this genus [5]. Chemical composition, biological activities, applications, and uses of different species of *Symphytum* are reported in the literature, whereas few are studies regarding the chemical composition of *S. anatolicum* [14,15]. Therefore, this investigation was aimed at providing a comprehensive phytochemical characterization of *S. anatolicum* whole plant, by an integrated LC-MS and ^1^H NMR metabolomic approach. Furthermore, phenolic content, antioxidant activity, glucosidase, and tyrosinase inhibitory activity of *S. anatolicum* MeOH extract were evaluated. The results of antioxidant and enzyme inhibitory assays suggested how *S. anatolicum* represents a source of bioactive compounds with health-promoting properties.

## 2. Materials and Methods

### 2.1. Chemical and Reagents

MeOH and water for HPLC were purchased from VWR (Milan, Italy). Acetonitrile, HCOOH, and water for LC-MS analysis were purchased from Merck (Merck KGaA, Darmstadt, Germany). MeOH-*d*_4_ (99.95%), D_2_O (99.9% containing 0.75 wt.% 3-(trimethylsilyl)propionic-2,2,3,3-d4, sodium salt), Folin–Ciocalteu, 1,1-diphenyl-2-picrylhydrazyl (DPPH), 6-hydroxy-2,5,7,8-tetramethylchroman-2-carboxylic acid (Trolox), diammonium 2,20-azinobis(3-ethylbenzothiazoline-6-sulfonate) (ABTS), quercetin 3-*O*-glucoside, vitamin C, kojic acid, L-tyrosine, acarbose and 4-nitrophenyl α-D-glucopyranoside (p-NPG) were purchased by Sigma-Aldrich (Milan, Italy). α-glucosidase from *Saccharomyces cerevisiae* and tyrosinase from mushrooms were purchased from Sigma-Aldrich (Milan, Italy).

### 2.2. Plant Material

Whole plants of *Symphytum anatolicum* Boiss. (Boraginaceae) were collected in Bozdağ (GPS coordinates latitude: 38.354274, longitude: 28.089896), Izmir, Turkey, after the beginning of the flowering period (April–May period). A voucher specimen was deposited in the Herbarium of Science Faculty, Ege University, Izmir, Turkey (EGE 12571).

### 2.3. General Procedures

Semi-preparative HPLC separations were carried out using a Symmetry Prep TM-C18 (78 mm × 300 mm, 7 μm) column on a Waters system (Milford, CT, USA) equipped with an R-401 refractive index detector and using a Phenomenex C18 Synergy-Hydro-RP (250 mm × 10 mm, 10 μm) column on an Agilent 1260 Infinity system (Agilent Technologies, Palo Alto, CA, USA), equipped with a binary pump (G-1312C) and a UV detector (G-1314B). TLC was performed on silica gel F254 (Macherey-Nagel Deltek, Naples, Italy) plates (20 cm × 10 cm), and mixtures of CHCl_3_–CH_3_OH–H_2_O (80:20:2, 70:30:3, 61:32:7) (VWR international PBI S.r.l., Milan, Italy) were used as mobile phases. Detection was carried out by spraying cerium (IV) sulphate followed by heating at 100 °C for 5 min. HR/ESI-MS data were acquired on an LTQ Orbitrap XL mass spectrometer (Thermo Fisher Scientific, Bremen, Germany) operating in negative-ion mode. A Thermo Scientific™ Multiskan SkyHigh Microplate Spectrophotometer was used for spectrophotometric assays.

### 2.4. LC-ESI/LTQOrbitrap/MS Analysis

LC-MS was performed using a system of Liquid Chromatography coupled to Electrospray Ionization and High-Resolution Mass Spectrometry (LC-ESI/HRMS) characterized by a quaternary Accela 600 pump and an Accela autosampler coupled to an LTQOrbitrap XL mass spectrometer (LTQ-Orbitrap XL, Thermo Fisher Scientific, Bremen, Germany) working in negative ion mode. A Phenomenex C18 Kinetex Evo-RP (150 mm × 2.1 mm, 5 µm) column at a flow rate of 0.2 mL/min was selected; water plus 0.1% formic acid and acetonitrile plus 0.1% formic acid were used as mobile phase A and B, respectively. A linear gradient method from 5 to 95% of B in 35 min was performed. The autosampler was set to inject 4 μL of methanol extract (1 mg/mL). The mass range was from *m*/*z* 120 to 1600 with a resolution of 30,000. A data-dependent scan experiment was performed for fragmentation, selecting precursor ions corresponding to the first and the second most intense ions from the LC-HRMS spectrum and using collision energy at 30%, a minimum signal threshold of 300, and an isolation width of 2.0.

### 2.5. Extraction and Isolation Procedures

Air-dried and powdered whole plant material (390 g) was extracted with hexane (2.5 L for two days), dichloromethane (2.5 L for two days), and methanol (2 × 2.5 L. each for two days) at room temperature (25 °C). After filtration and evaporation of solvent to dryness in vacuum, the dichloromethane (0.98 g) and methanol (12.64 g) extracts were obtained.

Two grams of MeOH extract were fractionated on a Sephadex LH-20 (GE Healthcare, Sigma Aldrich, Milan, Italy) column (100 cm × 5 cm), using MeOH as mobile phase, affording 186 fractions (8 mL), and monitored by TLC.

Further purifications were carried out by semipreparative HPLC-RI in isocratic conditions with MeOH-H_2_O at different percentages, at a flow rate of 2 mL/min. Fractions were dried and then dissolved in MeOH in a concentration of 10 mg/100 μL for the injections. The fractions 78–83 (71.8 mg) were chromatographed using MeOH–H_2_O (40:60) as mobile phase to produce compound **15** (1.4 mg, tR = 12.8 min). Fractions 84–103 (111.3 mg) were chromatographed using MeOH-H_2_O (40:60) as mobile phase to yield compounds **16** (1.2 mg, tR = 8.3 min), **11** (1.6 mg, tR = 11.6 min), **9** (1.8 mg, tR = 22.3 min). The fractions 116–128 (105.9 mg) were further chromatographed using MeOH-H_2_O (35:65) as mobile phase to produce compounds **7** (1.7 mg, tR = 32.1 min). The fractions 153–162 (24.9 mg) gave compound **17**. The fractions 181–186 (2.6 mg) gave compound **18**.

A total of 200 mg of MeOH extract was analyzed by an RP-HPLC-UV system. The mobile phase consisted of solvent A (H_2_O + 0.1% formic acid) and solvent B (CH_3_CN + 0.1% formic acid), at a flow rate of 2.0 mL/min. The HPLC gradient started at 5% B, after 5 min % B was 5%, after 15 min it was at 20%, after 20 min % B was at 50%, after 10 min it was at 100% as it was held for 5 min, to produce compounds **6** (1.1 mg, tR = 20.2 min), **3** (1.3 mg, tR = 23.1 min), **4** (2.4 mg, tR = 28.5 min), **5** (1.8 mg, tR = 29.9 min), **13** (1.4 mg, tR = 32.4 min), **8** (2.5 mg, tR = 32.8 min), **10** (2.4 mg, tR = 34.1 min), **14** (3.3 mg, tR = 36.4 min) and **12** (1.1 mg, tR = 39.7 min). The isolated compounds were injected into the LC-MS instrument and were used as standards, confirming their identification in the LC-MS profile of the extract (Table 1).

### 2.6. NMR Analysis and Data Processing

NMR experiments were acquired on a Bruker Ascend-600 NMR spectrometer (Bruker BioSpin GmBH, Rheinstetten, Germany) equipped with a Bruker 5 mm PATXI probe. DQF-COSY, HSQC, HMBC, and ROESY spectra were acquired in methanol-d4 (99.95%, Sigma-Aldrich), and standard pulse sequences and phase cycling were used. The 1D and 2D NMR data were processed by TOPSPIN 3.2 software.

For ^1^H NMR analysis, the extract (5.1 mg) was dissolved in 537 μL of phosphate buffer (1 M KH_2_PO_4_, D_2_O, and 2 mM NaN_3_ to prevent microbial contamination) after that, 13 μL of 44 mM TSP (trimethylsilyl propanoic acid) was added as internal standard and finally filled into a 5mm NMR tube. Experiments were run at 300 K in automation mode after loading individual samples on a Bruker Automatic Sample Changer, interfaced with the software IconNMR (Bruker). To suppress the residual water signal, a zgesgp Bruker standard pulse sequence was applied. A total of 80 transients (with 4 dummy scans) were collected into 64 k data points with a relaxation delay set to 5.0 s. The spectra were automatically Fourier transformed using an exponential window with a line broadening of 0.5 Hz. Phase and baseline were corrected using Chenomx NMR Suite 9.0 (Chenomx Inc., Edmonton, AB, Canada). Compounds were identified using the Chenomx and Human Metabolome Database (HMDB) 600 MHz libraries [17]. The metabolite concentrations were determined using the concentration of a known reference signal (TSP) [18].

### 2.7. Total Phenolic Content, DDPH, and TEAC Assays

Folin-Ciocalteu, DPPH, and TEAC (Trolox Equivalent Antioxidant Capacity) assays for the extract have been performed as previously reported [19]. The total phenolic content of the extracts was determined by Folin–Ciocalteu assay; gallic acid was used as a reference compound (calibration equation: y = 0.0027x + 0.0982, R^2^ = 0.9929). All the experiments were performed in triplicate, and results were expressed as the means of gallic acid equivalents (GAE mg/g dried extract).

For the TEAC assay, the extract was tested at concentrations of 250, 500, 750, and 1000 μg/mL. A total of 1.5 mL of diluted ABTS was added to 15 μL of each sample solution. The assay was carried out in triplicates. Quercetin 3-*O*-glucoside was used as reference standard. The results are presented as TEAC values, that is the concentration (mM) of a standard Trolox solution with the same antioxidant capacity as 1 μg/mL of the tested extract.

The stables radical DPPH^•^ was used to assess the antiradical activity of the extract following the procedure previously described [20]. Vitamin C was the positive control, while methanol was the negative control. A total of 37.5 μL of the methanol solution containing different concentrations of methanol extract has been added to 1.5 mL of daily prepared DPPH^•^ solution (0.025 g/L in MeOH). Absorbance has been measured at 517 nm 10 min after starting the reaction. The DPPH^•^ concentration in the reaction medium has been calculated from a calibration curve (range = 10–200 mg/mL) analyzed by linear regression (y = 0.257x + 5.55; R^2^ = 0.990). All experiments were conducted in triplicate.

### 2.8. α-Glucosidase Inhibition Assay

The α-glucosidase inhibitory activity was tested by using 96 well plates and following protocols previously reported [21]. The extract and acarbose were dissolved in MeOH and were tested at the final concentrations of 20 μg/mL, 8 μg/mL, 6 μg/mL, and 2 μg/mL. A total of 10 μL of each stock solution together with 15 μL of α-glucosidase from *Saccharomyces cerevisiae* (2 U/mL, 0.1 M potassium phosphate buffer, pH 6.8) were mixed in 150 μL of 0.1 M potassium phosphate buffer (pH 6.8). After 5 min of incubation at 37.0 °C, 75 μL of p-NPG (2.5 mM), as substrate, was added to the mixture, and the solution was incubated at 37.0 °C for another 10 min. The absorbance of the generated p-nitrophenol was measured at 405 nm using a UV spectrometer. The sample was replaced with 10 μL of buffer (potassium phosphate buffer) for the negative control. The experiment was conducted in triplicate. The IC_50_ value (μg/mL) represents the concentration inhibiting the α-glucosidase activity by 50%.

The α-glucosidase inhibitory activity was calculated by using the following formula:

inhibition (%): [(XA − XB)/XA] × 100(1)

XA is the absorbance of the control and XB is the absorbance of the sample.

### 2.9. Tyrosinase Inhibition Assay

The tyrosinase inhibitory activity was evaluated using a method described by Oh et al. [22]. 30 microliters of the sample (300, 100, 50, and 25 µg/mL), and 50 µL of 100 U/mL mushroom tyrosinase were treated in 96-well plates and incubated at 37 °C for 15 min. Subsequently, 50 µL of 1 mM L-tyrosine was added and then reacted at 37 °C for 15 min. The amount of dopachrome formed was measured at 495 nm. Each sample has been tested in triplicate and the tyrosinase inhibitory activity was calculated using the following equation:

tyrosinase inhibition (%) = [1 − (S − S0)/(C − C0)] × 100(2)

where S is the absorbance of the sample, tyrosinase, and L-tyrosine; S0 is the absorbance of the sample and L-tyrosine; C is the absorbance of tyrosinase and L-tyrosine, and C0 is the absorbance of L-tyrosine. Kojic acid, a known tyrosinase inhibitor, was used as a positive control.

### 2.10. Statistical Analysis of Data

Data are reported as means ± mean standard error (SEM) of at least three independent experiments. Each experiment was conducted in triplicate. A *p*-value less than 0.05 was considered significant.

## 3. Results

### 3.1. LCESI/Orbitrap/MS/MS Analysis of MeOH extract of S. anatolicum

**Figure 1:**
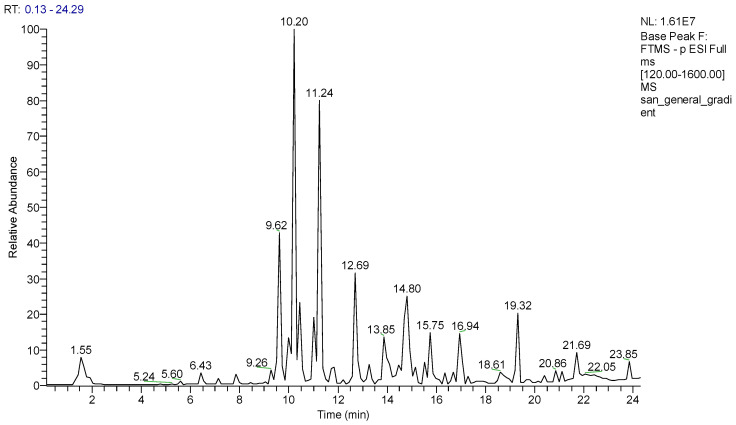
LC-ESI-FT-MS profile of *Symphytum anatolicum* methanol extract in negative ion mode.

The methanol extract of *S. anatolicum* was investigated by preliminary analysis using an extremely sensitive analytical technique such as liquid chromatography coupled to high-resolution tandem mass spectrometry in negative ionization mode, using a mass spectrometer with electrospray source coupled to Orbitrap mass analyzer (LC-ESI-FT-MS). The LC-ESI-FT-MS profile (Figure 1) showed the presence of 21 main specialized metabolites, belonging to the classes of flavonoids, phenylpropanoids, salvianols, and oxylipins. Initially, some peaks were tentatively identified, based on their accurate masses, on the characteristic fragmentation pattern, and by comparing the results obtained with the data reported in the literature on *S. anatolicum* and in databases (database “KNApSAcK”). Compound **2** showed a precursor ion [M-H]^−^ at *m*/*z* 377.0857, supporting the molecular formula C_18_H_18_O_9_. Comparing the results of mass spectra with the library and the literature data, compound **2** was identified as dan shen suan C. Compounds **3** and **4** have been identified as 2,5-dihydroxybenzoic acid and 4-hydroxybenzoic acid, already reported in *S. anatolicum* [14]. The analysis of the fragmentation spectra of compounds **7**–**11** made it possible to ascertain the presence of sugar units in different positions of the aglycone, determining characteristic neutral losses from precursor ions of 162 and 132 Da, corresponding to the dehydrated form of hexose and pentose, respectively, allowing to identify the compounds as kaempferol or quercetin glycosides. Compounds **5**, **7**, **8**, **10**, and **14** had already been reported in the species *S. anatolicum* [15] and were identified as caffeic acid (**5**) (C_9_H_8_O_4_), rutin (**7**) (C_27_H_30_O_16_), quercetin-3-*O*-glucoside (**8**) (C_21_H_20_O_12_), kaempferol 3-*O*-glucoside (**10**) (C_21_H_20_O_11_), rosmarinic acid (**14**) (C_18_H_16_O_8_). The molecular formula together with the fragmentation spectra of compounds **15**–**18** allowed us to identify them as salvianolic acid A (C_26_H_22_O_10_) and C (C_26_H_20_O_10_) (**15**,**16**), previously reported in *S. anatolicum*, and salvianolic acid B (**17**) (C_36_H_30_O_16_), previously reported in *S. officinale* [15,23]. Compound **18** showed a precursor ion [M-H]^−^ at *m*/*z* 551.1200 supporting a molecular formula C_28_H_24_O_12_; the fragmentation pattern was similar to those of salvianolic acids with the presence of fragment ions at *m*/*z* 519.0928 corresponding to the neutral loss of 32 Da assigned as MeOH unit, allowing us to suppose esterification of the carboxyl group in the furan ring of lithospermic acid, and at *m*/*z* 339.0498, corresponding to the neutral loss of 180 Da assigned as caffeic acid unit. Thus compound **18** was identified as monomethyl lithospermate, for the first time reported in *Symphytum* species. Salvianolic acids can be divided into monomers and polymers (dimers, trimers, and tetramers) based on the number of phenyls present in their structures [24]. In general, the polymers of salvianolic acids generate product ions in the tandem mass spectra due to successive or simultaneous loss of C_9_H_6_O_3_ (162.0317 Da), C_9_H_8_O_4_ (180.0423 Da) and C_9_H_10_O_5_ (198.0528 Da), corresponding respectively to caffeoyl unit, caffeic acid or to the loss of dan shen suan C. Compounds **19**–**21** showed a typical fragmentation pattern ascribable to oxylipins, fatty acids differing from each other in the degree of unsaturation and number of hydroxyl groups. The product ions generated by one or more consecutive neutral losses of 18 Da allowed us to define the number of hydroxyl groups present in the oxylipine structure. By detecting characteristic product ions, such as those at *m*/*z* 171 (C_9_H_15_O_3_) and *m*/*z* 201 (C_10_H_17_O_4_), or those at *m*/*z* 253 (C_15_H_25_O_3_), 229 (C_12_H_21_O_4_), 223 (C_14_H_23_O_2_) and 199 (C_11_H_19_O_3_), these hydroxyl groups could be located at the head (precisely at the C9 and C10 positions) or at the tail of the oxylipin (precisely at C12, C13, C15 and C16) [25]. Furthermore, along with the molecular formula and RDB (Double Bond/Ring Equivalents) value, the fragmentation pattern allowed in some cases to establish the position of the double bond. In this way, the compounds were identified as those reported in Table 1. In order to accurately determine the structures of the compounds present in the methanol extract of *S. anatolicum*, the extract was purified by chromatography, using Sephadex LH-20, HPLC-UV, and HPLC-RI. In detail, the methanol extract was first fractionated by size exclusion chromatography on Sephadex LH-20, and the obtained fractions were further purified by HPLC; for some fractions, a refractive index (RI) detector was used, and for some other fractions a UV detector.

**Figure 2:**
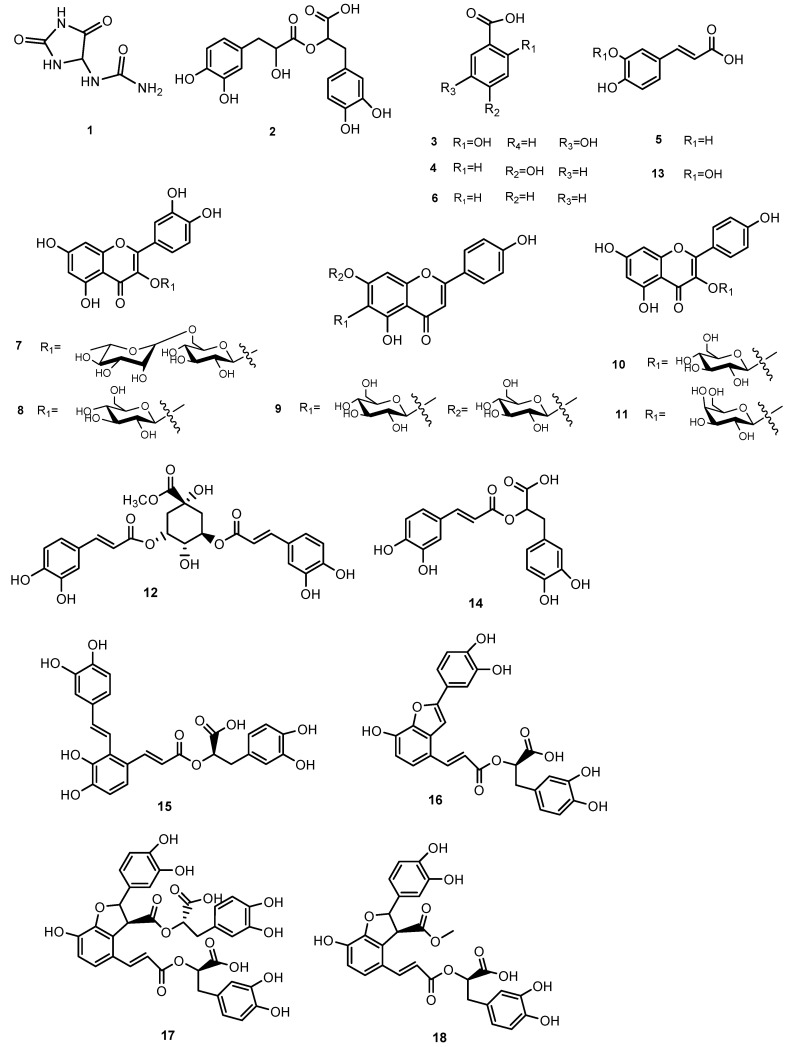
Compounds occurring in *Symphytum anatolicum* methanol extract.

The isolated compounds have been shown in Table 1 (Figure 2).

### 3.2. NMR Analysis of MeOH Extract of S. anatolicum

**Figure 3:**
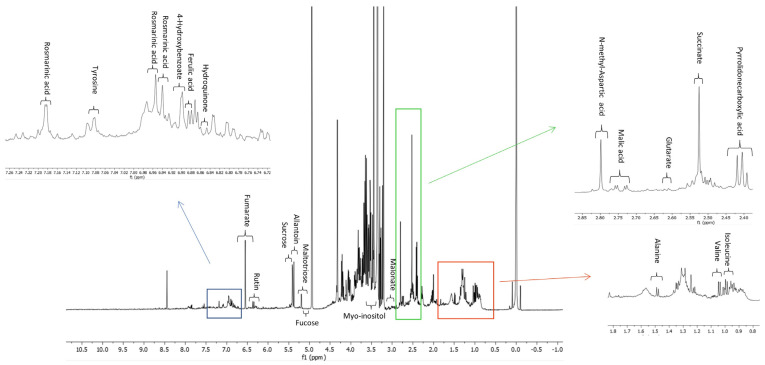
^1^H NMR spectrum of *Symphytum anatolicum* methanol extract.

To obtain complete information not only about the occurrence of specialized metabolites but also of primary metabolites, the MeOH extract of *S. anatolicum* was investigated by an approach based on ^1^H NMR analysis [19,20,26]. NMR is one of the most useful analytical techniques which can contribute to the field of metabolomics. The ^1^H NMR spectrum of the extract showed the presence of various classes of metabolites. NMR has a universal detection capability, since any molecule containing one or more atoms with a non-zero magnetic moment (such as ^1^H, ^13^C, ^14^N, 15N, and ^31^P), is potentially detectable by NMR, providing unbiased detections and covering compounds of all organic chemical classes in plant extracts. ^1^H NMR is a non-destructive technique and is characterized by a simple sample preparation and rapid analysis. It can simultaneously identify diverse groups of specialized metabolites as well as abundant primary compounds [26]. A comprehensive inspection of the ^1^H NMR spectrum based on its characteristic signals allowed the identification of organic acids, phenolics, flavonoids, sugars, and amino acids. A total of 21 metabolites were identified by comparison with the literature data, the Chenomx database, and on the basis of 2D NMR spectra. The identified metabolites are displayed in Figure 3. The most intense signals were observed in the region ranging from 3.00 to 5.00 ppm, representing the carbohydrate constituents (sucrose, fucose, and maltotriose) [27]. In the low-frequency region, characteristic peaks of the amino acids alanine, valine, and isoleucine were detected while other two amino acids, tyrosine, and N-methyl-aspartic acid were identified in the mid to high-frequency region (Figure 3).

Along with primary metabolites, the occurrence of specialized metabolites was revealed. For the complete and unambiguous assignment of some compounds, standard two-dimensional NMR experiments, namely COSY, TOCSY, and HSQC, were performed. To make some identifications and assignments more robust, the obtained results were integrated with the information provided by the reference library of Chenomx and the NMR spectra available in the Human Metabolome Database [18,28].

The principal peak areas of the spectrum afforded an immediate measure of metabolite concentration allowing all the metabolites to be quantified based on a single internal standard. Indeed, the concentration of the identified metabolites was obtained, with respect to the known concentration of TSP, by means of the well-established software package, Chenomx [29]. This latter software is based on a highly sophisticated targeted profiling technology that allows an easy deconvolution of complex NMR spectra into their individual components that can be quantified with good accuracy [18].

The metabolites average molar concentrations, expressed both as mM and as mg/g MeOH extract, showed a high sugar content (sucrose, fucose, and maltotriose with a concentration of 10.13, 8.25, and 19.06 mg/g extract, respectively). Allantoin, a metabolite deriving from uric acid oxidation [30], was identified in *S. officinale* and *S. cordatum* extracts [5]. Allantoin is a safe and non-toxic compound, used in cosmetics for its soothing, skin softening, cell proliferation stimulating, and injured tissue repairing properties [5]. Overall, allantoin is a versatile ingredient with a range of biological effects such as wound healing, anti-inflammatory, antioxidant, and antidiabetic [31,32]. Herein, the MeOH extract of *S. anatolicum* revealed a content in allantoin of 2.47 mg/g extract.

Regarding specialized metabolites, the extract appeared a good source of rosmarinic acid (3.18 mg/g extract) as well as of rutin (8.58 mg/g extract).

### 3.3. Evaluation of Phenolic Content and Antioxidant Activity

Considering the chemical profile of the extract, the phenolic content was determined by the Folin-Ciocalteu method [26], before evaluating the radical scavenging activity. The result of the total phenolic content determination indicated a phenolic content, expressed as milligrams of gallic acid equivalents per gram of extract, of 21.40 mg GAE/g (±0.35). Successively, the antioxidant activity of *S. anatolicum* extract was tested by TEAC and DPPH assays. The methanol extract displayed a radical scavenging activity (TEAC value of 1.80 ± 0.05 mM) higher than that of quercetin 3-*O*-glucoside (0.69 ± 0.05 mM), used as a reference compound. The DPPH assay displayed antioxidant activity with an EC_50_ value of 115.7 ± 0.93 µg/mL). Ascorbic acid was used as a reference compound (EC_50_ = 4.4 ± 0.03 µg/mL).

### 3.4. α-Glucosidase Inhibitory Activity

*Symphytum* is a medicinal herb that has been used for centuries to treat a variety of ailments, including diabetes. This species contains several active compounds such as allantoin and rosmarinic acid, which have been shown to have antidiabetic effects [5,33]. The antidiabetic effects of *Symphytum* species like *S. officinale* and *S. anatolicum* have been extensively studied, showing high α-glucosidase inhibitory activity [15,34]. The inhibitory effect of the extract of the whole plant of *S. anatolicum* was evaluated against α-glucosidase and showed a potent inhibitory activity (IC_50_ = 18.28 ± 0.31 μg/mL), comparable to that exerted by acarbose (IC_50_ = 17.05 ± 0.25 μg/mL), used as a positive control.

### 3.5. Tyrosinase Inhibitory Activity

Tyrosinase is an enzyme that plays a key role in melanin synthesis. It catalyzes two distinct reactions occurring during this process: the hydroxylation of tyrosine and the oxidation of 3,4-dihydroxyphenylalanine (L-DOPA) to o-dopaquinone [35,36]. It is reported that the abnormal and uncontrolled production and distribution of melanin can cause many dermatological disorders, such as lentigines, melasma, and post-inflammatory hyperpigmentation [36,37]. In recent years, there has been a growing interest in the search for new tyrosinase inhibitors [38]. Herein, *S. anatolicum* extract was investigated for its potential inhibitory activity against mushroom tyrosinase. Results showed that MeOH extract presented an interesting tyrosinase inhibitory activity (IC_50_ = 20.59 ± 0.30 µg/mL). Kojic acid, considered a strong inhibitor of tyrosinase, was used as a reference (IC_50_ = 23.18 ± 0.35 µg/mL).

## 4. Discussion

The comprehensive investigation of *S. anatolicum* whole plant extract was performed by LC-MS and NMR analysis to highlight the occurrence of both specialized and primary metabolites. The LC-MS analysis, more sensitive than the NMR, allowed us to identify 21 phenolic compounds, belonging to phenolic acids, flavonoids, and lignans.

Rosmarinic acid (**14**) corresponded to one of the highest peaks observed in the LC-MS profile, in agreement with previous investigations on *S. anatolicum* and also on *Symphytum* genus [5,14,15]. Compared to the previous investigation reported for *S. anatolicum* [14,15], allantoin (**1**), dan shen suan C (**2**), benzoic acid (**6**), saponarin (**9**), 3,5-dicaffeoylquinic acid (3,5-*O*-DCQA, **12**), monomethyl lithospermate (**18**) and oxylipins (**19**–**21**) are reported here for the first time in the species. Among these compounds, allantoin is considered a marker of comfrey, and monomethyl lithospermate (**18**) was previously reported in *Arnebia radix* and *Lithospermum erythrorhizon*, belonging to the Boraginaceae family [39,40].

Regarding allantoin (**1**), although reported as a main compound in the *Symphytum* genus [5], it did not appear as a high peak in the LC-MS profile acquired in negative ionization mode. Its relevant content, reported as responsible for the wound-healing property of *Symphytum* spp., was evident in the NMR analysis (Figure 3 and Table 2) which, compared to the LC-MS analysis, evidenced primary metabolites as amino acids, sugars, and organic acids. Among amino acids, N-methyl-aspartic acid was previously described by GC-MS in the fruits of *Lycium chinense* and *Lycium barbarum*, also known as Lycii Fructus [40].

Moreover, rutin (**7**) and rosmarinic acid (**14**), identified by LC-MS analysis, were also evident in the NMR profile. These results highlight how the two analytical techniques, LC-MS and NMR, afford complementary information that combined offer an in-depth knowledge of the plant metabolome.

In this work, NMR analysis allowed also the quantitative analysis of *S. anatolicom* methanol extract. NMR analyses provide direct quantitative information because the intensity of the proton signal is proportional to the molar concentration of the metabolites. In previous studies, Sarikurkcu et al. reported the quantitative analysis of phenolic compounds in the methanol extract of *S. anatolicum* roots by an LC-MS approach using the Multiple Reaction Monitoring (MRM) experiments [14]; rosmarinic acid represented one of the major compounds isolated, in agreement with the literature data for the comfrey roots [5]. Taking into account that the plant material was the whole plant, the quantitative analysis of rosmarinic acid in the methanol extract of *S. anatolicum* was comparable to that reported by Sarikurkcu [14].

The phenolic content, expressed as mg GAE/g of extract, was in agreement with that reported for the aerial parts and the roots of *S. anatolicum* [14,15]. DPPH and ABTS assays revealed a significant antioxidant activity. Furthermore, comparing our results with the literature, it was observed that methanol extract from the whole plant of *S. anatolicum* showed higher DPPH assay than the methanol extract of the roots [14].

Enzyme inhibitory surveys, such as inhibitors of α-glucosidase and tyrosinase, are regarded as key strategies for reducing the risk of many severe health problems. So, α-glucosidase and tyrosinase inhibitory activity was tested for the methanol extract of the whole plant. Our results showed that the methanol extract of the whole plant presented a higher tyrosinase inhibitory activity than the roots [14]. This could be attributed to the fact that different molecules are biosynthesized from different plant organs, so the inhibitory activity depends on the chemical profile of each raw material.

## 5. Conclusions

Considering the innumerable potentialities of the *Symphytum* species and their widespread use in the world, it is extremely important to define their phytochemical profile and bioactivity. The metabolome varies among species, and organs, due to genetic and environmental factors, and its investigation may provide better mechanistic understanding in ecological and evolutionary contexts. The metabolome lies at the interface between genes and the environment, and for this reason, by comprising the composition, abundance, and interplay of many thousands of metabolites, it provides a direct and multidimensional measure of the molecular mechanisms through which evolutionary and ecological processes shape plant functioning [1]. Over recent decades, advances in analytical methods have led to the advent of metabolomics, and each of these shows advantages and disadvantages.

^1^H NMR can provide comprehensive characteristic fingerprints of herbal products and it is widely used to analyze plant metabolites [20]. Noteworthy, a combined approach of NMR and LC-MS analysis provides a comprehensive phytochemical investigation of *S. anatolicum*.

The results of the antioxidant and enzyme inhibitory assays of the present study demonstrate the methanol extract of *S. anatolicum* as a source of bioactive compounds with health-promoting properties. These findings provide promising evidence for further investigation into the specific bioactive compounds responsible for the α-glucosidase and tyrosinase inhibitory effects. Therefore, the methanol extract of *S. anatolicum* shows a significant therapeutic potential for the treatment of metabolic and dermatological disorders.

## Figures and Tables

**Table 1:** Compounds identified in *Symphytum anatolicum* extract by LC-ESI-FT-MS, LC-ESI-FT-MS/MS analysis, and NMR spectroscopy.

N°	Rt	[M-H]^−^	Molecular Formula	ppm	MS/MS	Identity
**1**	1.54	157.0362	C_4_H_6_O_3_N_4_	−0.63	140.01/114.03/97.00	Allantoin *
**2**	1.86	377.0857	C_18_H_18_O_9_	−2.6	197.0471	dan shen suan C *
**3**	4.40	153.0184	C_7_H_6_O_4_	1.9	-	2,5-dihydroxybenzoic acid
**4**	5.60	137.0240	C_7_H_6_O_3_	1.4	-	4-hydroxybenzoic acid
**5**	7.14	179.0346	C_9_H_8_O_4_	1.1	-	caffeic acid
**6**	7.26	121.0301	C_7_H_6_O_2_	3.0	-	benzoic acid
**7**	9.26	609.1460	C_27_H_30_O_16_	0.8	301.0356	rutin
**8**	9.62	463.0879	C_21_H_20_O_12_	0.7	301.0341	quercetin-3-*O*-glucoside
**9**	9.98	593.1509	C_27_H_30_O_15_	0.5	285.0405	saponarin
**10**	10.20	447.0930	C_21_H_20_O_11_	0.67	285.0407	kaempferol 3-*O*-glucoside
**11**	10.43	447.1348	C_15_H_28_O_15_	−0.44	285.0407	kaempferol 3-*O*-galactoside
**12**	10.62	515.1190	C_25_H_24_O_12_	0.19	353.0872	3,5-*O*-DCQA
**13**	11.12	193.0505	C_10_H_10_O_4_	2.5	-	ferulic acid
**14**	11.24	359.0770	C_18_H_16_O_8_	1.11	161.0249/179.0359/197.0472	rosmarinic acid
**15**	11.85	493.1136	C_26_H_22_O_10_	0.40	295.0613	salvianolic acid A
**16**	12.69	491.0980	C_26_H_20_O_10_	0.40	311.0564	salvianolic acid C
**17**	12.80	717.1460	C_36_H_30_O_16_	0.69	519.0928/339.0498/321.0403	salvianolic acid B
**18**	13.23	551.1200	C_28_H_24_O_12_	2.8	519.0922/371.0768/353.0666/339.0506	monomethyl lithospermate
**19**	13.85	327.2180	C_18_H_32_O_5_	2.7	309.2076/291.1965/211.1346/171.1030	9,12,13-TriHODE (10,15) *
**20**	14.76	329.2333	C_18_H_34_O_5_	1.51	171.1030/229.1445/293.2128/311.2228	9,12,13 triHOME (10) *
**21**	15.15	327.2179	C_18_H_32_O_5_	2.5	229.1445/211.1346/171.1030/291.1965	Oxo-9,10DiHOME (11) *

* Compounds detected and identified by LC-MS analysis.

**Table 2:** Metabolites average molar concentrations in *S. anatolicum* methanol extract.

	mM	mg/g MeOH Extract
Amino acids		
Alanine	0.0148 ± 0.0052	0.1293 ± 0.0454
Isoleucine	0.0254 ± 0.0085	0.3266 ± 0.1093
Tyrosine	0.1716 ± 0.0018	3.0483 ± 0.0320
N-Methyl-aspartic acid	0.2474 ± 0.0349	3.5686 ± 0.5034
Valine	0.0424 ± 0.0037	0.4870 ± 0.0425
Sugars		
Sucrose	0.3021 ± 0.0028	10.1381 ± 0.0940
Fucose	0.5127 ± 0.0374	8.2515 ± 0.6019
Maltotriose	0.3855 ± 0.0293	19.0634 ± 1.4489
Organic acids		
Pyrrolidonecarboxylic acid	0.3492 ± 0.0975	4.4201 ± 1.2341
Succinic acid	0.1020 ± 0.0154	1.1607 ± 0.1752
4-Hydroxybenzoic acid	0.0129 ± 0.0052	0.1734 ± 0.0699
Malonic acid	0.3249 ± 0.0287	3.2506 ± 0.2871
Glutaric acid	0.0509 ± 0.0200	0.6543 ± 0.2571
Fumaric acid	0.0663 ± 0.0023	0.7414 ± 0.0257
Malic acid	0.0986 ± 0.0251	1.2962 ± 0.3300
Phenolic compounds		
Rutin	0.1435 ± 0.0152	8.5889 ± 0.9098
Rosmarinic acid	0.0900 ± 0.0062	3.1791 ± 0.2190
3,4-dihydroxyhydrocinnamic acid	0.0934 ± 0.0063	1.6482 ± 0.1112
Others		
Myo-inositol	0.2131 ± 0.0999	3.7639 ± 1.7645
Hydroquinone	0.0160 ± 0.0021	0.1727 ± 0.0227
Allantoin	0.1595 ± 0.0237	2.4726 ± 0.3674

The concentration of the identified metabolites was obtained, with respect to the known concentration of TSP, by means of Chenomx.

## Data Availability

The data presented in this study are available in the main article.

## References

[1] Walker T.W.N., Alexander J.M., Allard P.-M., Baines O., Baldy V., Bardgett R.D., Capdevila P., Coley P.D., David B., Defossez E. (2022). Functional Traits 2.0: The power of the metabolome for ecology. J. Ecol..

[2] Ikhalaynen Y.A., Plyushchenko I.V., Rodin I.A. (2022). Hopomics: *Humulus lupulus* Brewing Cultivars Classification Based on LC-MS Profiling and Nested Feature Selection. Metabolites.

[3] Ivanović S., Simić K., Lekić S., Jadranin M., Vujisić L., Gođevac D. (2022). Plant Metabolomics as a Tool for Detecting Adulterants in Edible Plant: A Case Study of *Allium ursinum*. Metabolites.

[4] Zhao J.P., Wang M., Saroja S.G., Khan I.A. (2022). NMR technique and methodology in botanical health product analysis and quality control. J. Pharm. Biomed. Anal..

[5] Salehi B., Sharopov F., Tumer T.B., Ozleyen A., Rodriguez-Perez C., Ezzat S.M., Azzini E., Hosseinabadi T., Butnariu M., Sarac I. (2019). *Symphytum* Species: A Comprehensive Review on Chemical Composition, Food Applications and Phytopharmacology. Molecules.

[6] Horinouchi C.D.S., Otuki M.F. (2013). Botanical Briefs: Comfrey (*Symphytum officinale*). Cutis.

[7] Trifan A., Zengin G., Sinan K.I., Esslinger N., Grubelnik A., Wolfram E., Skalicka-Woźniak K., Minceva M., Luca S.V. (2021). Influence of the Post-Harvest Storage Time on the Multi-Biological Potential, Phenolic and Pyrrolizidine Alkaloid Content of Comfrey (*Symphytum officinale* L.) Roots Collected from Different European Regions. Plants.

[8] Hacioglu B.T., Erik S. (2011). Phylogeny of *Symphytum* L. (Boraginaceae) with special emphasis on Turkish species. Afr. J. Biotechnol..

[9] Nastić N., Borrás-Linares I., Lozano-Sánchez J., Švarc-Gajić J., Segura-Carretero A. (2020). Comparative Assessment of Phytochemical Profiles of Comfrey (*Symphytum officinale* L.) Root Extracts Obtained by Different Extraction Techniques. Molecules.

[10] Rode D. (2002). Comfrey toxicity revisited. Trends Pharmacol. Sci..

[11] Seigner J., Junker-Samek M., Plaza A., D’Urso G., Masullo M., Piacente S., Holper-Schichl Y.M., de Martin R. (2019). A *Symphytum officinale* Root Extract Exerts Anti-inflammatory Properties by Affecting Two Distinct Steps of NF-kappa B Signaling. Front. Pharmacol..

[12] Sharma A., Khanna S., Kaur G., Singh I. (2021). Medicinal plants and their components for wound healing applications. Future J. Pharm. Sci..

[13] Staiger C. (2012). Comfrey: A Clinical Overview. Phytother. Res..

[14] Sarikurkcu C., Ozer M.S., Tlili N. (2019). LC–ESI–MS/MS characterization of phytochemical and enzyme inhibitory effects of different solvent extract of *Symphytum anatolicum*. Ind. Crop. Prod..

[15] Varvouni E.-F., Zengin G., Graikou K., Ganos C., Mroczek T., Chinou I. (2020). Phytochemical analysis and biological evaluation of the aerial parts from *Symphytum anatolicum* Boiss. and *Cynoglottis barrelieri* (All.) Vural & Kit Tan (Boraginaceae). Biochem. Syst. Ecol..

[16] Neagu E., Paun G., Albu C., Eremia S.A.-M.V., Radu G.L. (2023). *Artemisia abrotanum* and *Symphytum officinale* Polyphenolic Compounds-Rich Extracts with Potential Application in Diabetes Management. Metabolites.

[17] Cicero N., Corsaro C., Salvo A., Vasi S., Giofre S.V., Ferrantelli V., Di Stefano V., Mallamace D., Dugo G. (2015). The metabolic profile of lemon juice by proton HR-MAS NMR: The case of the PGI Interdonato Lemon of Messina. Nat. Prod. Res..

[18] Cerulli A., Masullo M., Montoro P., Hosek J., Pizza C., Piacente S. (2018). Metabolite profiling of “green” extracts of *Corylus avellana* leaves by H-1 NMR spectroscopy and multivariate statistical analysis. J. Pharm. Biomed. Anal..

[19] Cerulli A., Masullo M., Piacente S. (2021). Metabolite Profiling of Helichrysum italicum Derived Food Supplements by H-1-NMR-Based Metabolomics. Molecules.

[20] Kilinc H., Masullo M., Lauro G., D’Urso G., Alankus O., Bifulco G., Piacente S. (2023). *Scabiosa atropurpurea*: A rich source of iridoids with-glucosidase inhibitory activity evaluated by in vitro and in silico studies. Phytochemistry.

[21] Oh K.E., Shin H., Lee M.K., Park B., Lee K.Y. (2021). Characterization and Optimization of the Tyrosinase Inhibitory Activity of Vitis amurensis Root Using LC-Q-TOF-MS Coupled with a Bioassay and Response Surface Methodology. Molecules.

[22] Trifan A., Opitz S.E.W., Josuran R., Grubelnik A., Esslinger N., Peter S., Bram S., Meier N., Wolfram E. (2018). Is comfrey root more than toxic pyrrolizidine alkaloids? Salvianolic acids among antioxidant polyphenols in comfrey (*Symphytum officinale* L.) roots. Food Chem. Toxicol..

[23] Wang J., Xu J., Gong X., Yang M., Zhang C., Li M. (2019). Biosynthesis, Chemistry, and Pharmacology of Polyphenols from Chinese Salvia Species: A Review. Molecules.

[24] Napolitano A., Cerulli A., Pizza C., Piacente S. (2018). Multi-class polar lipid profiling in fresh and roasted hazelnut (*Corylus avellana* cultivar “Tonda di Giffoni”) by LC-ESI/LTQOrbitrap/MS/MS(n). Food Chem..

[25] Cerulli A., Masullo M., Pizza C., Piacente S. (2022). Metabolite Profiling of “Green” Extracts of *Cynara cardunculus* subsp. *scolymus*, Cultivar “Carciofo di Paestum” PGI by H-1 NMR and HRMS-Based Metabolomics. Molecules.

[26] Borim de Souza A.J., Ocampos F.M.M., Catoia Pulgrossi R., Dokkedal A.L., Colnago L.A., Cechin I., Saldanha L.L. (2023). NMR-Based Metabolomics Reveals Effects of Water Stress in the Primary and Specialized Metabolisms of *Bauhinia ungulata* L. (Fabaceae). Metabolites.

[27] Abdul Hamid N.A., Mediani A., Maulidiani M., Abas F., Park Y.S., Leontowicz H., Leontowicz M., Namiesnik J., Gorinstein S. (2017). Characterization of metabolites in different kiwifruit varieties by NMR and fluorescence spectroscopy. J. Pharm. Biomed. Anal..

[28] Corol D.I., Harflett C., Beale M.H., Ward J.L. (2014). An Efficient High Throughput Metabotyping Platform for Screening of Biomass Willows. Metabolites.

[29] Ohyama T., Isaka M., Saito A., Higuchi K. (2023). Effects of Nodulation on Metabolite Concentrations in Xylem Sap and in the Organs of Soybean Plants Supplied with Different N Forms. Metabolites.

[30] Selamoglu Z., Dusgun C., Akgul H., Gulhan M.F. (2017). In-Vitro Antioxidant Activities of the Ethanolic Extracts of Some Contained-Allantoin Plants. Iran. J. Pharm. Res..

[31] Dawood M.F.A., Tahjib-Ul-Arif M., Sohag A.A., Latef A.A.H.A., Ragaey M.M. (2021). Mechanistic Insight of Allantoin in Protecting Tomato Plants against Ultraviolet C Stress. Plants.

[32] Bao T.Q., Li Y., Qu C., Zheng Z.G., Yang H., Li P. (2020). Antidiabetic Effects and Mechanisms of Rosemary (*Rosmarinus officinalis* L.) and its Phenolic Components. Am. J. Chin. Med..

[33] Chen S.L., Shang H.M., Yang J.Y., Li R., Wu H.X. (2018). Effects of different extraction techniques on physicochemical properties and activities of polysaccharides from comfrey (*Symphytum officinale* L.) root. Ind. Crop. Prod..

[34] Pillaiyar T., Manickam M., Namasivayam V. (2017). Skin whitening agents: Medicinal chemistry perspective of tyrosinase inhibitors. J. Enzym. Inhib. Med. Chem..

[35] Zaidi K.U., Ali S.A., Ali A., Naaz I. (2019). Natural Tyrosinase Inhibitors: Role of Herbals in the Treatment of Hyperpigmentary Disorders. Mini Rev. Med. Chem..

[36] Shenoy A., Madan R. (2020). Post-Inflammatory Hyperpigmentation: A Review of Treatment Strategies. J. Drugs Dermatol..

[37] Zhang X.W., Bian G.L., Kang P.Y., Cheng X.J., Yan K., Liu Y.L., Gao Y.X., Li D.Q. (2021). Recent advance in the discovery of tyrosinase inhibitors from natural sources via separation methods. J. Enzyme Inhib. Med. Chem..

[38] Zhu L., Ma S., Li K., Xiong P., Qin S., Cai W. (2022). Systematic Screening of Chemical Constituents in the Traditional Chinese Medicine Arnebiae Radix by UHPLC-Q-Exactive Orbitrap Mass Spectrometry. Molecules.

[39] Thuong P.T., Kang K.W., Kim J.K., Seo D.B., Lee S.J., Kim S.H., Oh W.K. (2009). Lithospermic acid derivatives from *Lithospermum erythrorhizon* increased expression of serine palmitoyltransferase in human HaCaT cells. Bioorg. Med. Chem. Lett..

[40] Ryu M.J., Kim M., Ji M., Lee C., Yang I., Hong S.B., Chin J., Seo E.K., Paik M.J., Lim K.M. (2020). Discrimination of *Lycium chinense* and *L. barbarum* Based on Metabolite Analysis and Hepatoprotective Activity. Molecules.

